# Subtenon Autologous Platelet-Rich Plasma in Degenerative Retinal Diseases: A Prospective Pilot Study of Safety and Exploratory Functional Signals in Retinitis Pigmentosa and EMAP

**DOI:** 10.3390/biomedicines14051029

**Published:** 2026-04-30

**Authors:** Rubens Camargo Siqueira, Cinara Cássia Brandão, Andreia Conceição de Jesus Souza, Juliana Rodrigues Seixas, Marisa Aparecida Balbino, Luma Moreira Antunes, Charles Muniz de Oliveira, Tainara Souza Pinho, Patrícia Fischer Cruz

**Affiliations:** 1 Rubens Siqueira Research Center, São José do Rio Preto 15010-100, SP, Brazil; 2 Postgraduate Department of the Medical School of São José do Rio Preto, Faculdade de Medicina de São José do Rio Preto—FAMERP, São José do Rio Preto 15090-000, SP, Brazil; 3 Immunogenetics Laboratory, Department of Dermatological, Infectious and Parasitic Diseases, Faculdade de Medicina de São José do Rio Preto—FAMERP, São José do Rio Preto 15090-000, SP, Brazil; 4 Criovida—Cell Biotechnology Center, Fleury Group, Belo Horizonte 30455-610, MG, Brazil

**Keywords:** platelet-rich plasma, subtenon injection, retinitis pigmentosa, extensive macular atrophy with pseudodrusen-like appearance (EMAP), degenerative retinal diseases, neuroprotection, immunomodulation, regenerative therapy

## Abstract

**Purpose:** To evaluate the safety and feasibility of repeated subtenon administration of autologous platelet-rich plasma (PRP) in patients with degenerative retinal diseases and to explore preliminary, hypothesis-generating functional observations in retinitis pigmentosa (RP) and extensive macular atrophy with pseudodrusen-like appearance (EMAP). **Methods:** This prospective, open-label, uncontrolled pilot study included 13 patients (6 RP, 7 EMAP) who received three subtenon PRP injections (1.5 mL each) at baseline, Month 2, and Month 4, with follow-up through Month 6. The study was designed primarily to assess safety and feasibility and was not powered or intended to evaluate efficacy. The primary outcome was safety, including adverse events and intraocular pressure changes. Exploratory secondary outcomes included best-corrected visual acuity (BCVA, logMAR), visual field mean deviation (MD), and structural optical coherence tomography (OCT) parameters. Electrophysiological outcomes were analyzed descriptively due to incomplete paired data. Analyses were conducted within diagnostic groups, and no between-group comparisons were performed. **Results:** All 13 patients completed the study. No serious adverse events or permanent ocular morbidity were observed. Two transient and self-limited adverse events occurred (anterior uveitis and intraocular pressure elevation), both resolving without sequelae. In the overall cohort, BCVA remained stable without statistically significant change. In the RP subgroup, a small exploratory change in BCVA was observed (mean ΔlogMAR −0.09; nominal *p* = 0.048), corresponding to approximately 4–5 ETDRS letters; however, this finding was associated with wide confidence intervals and limited statistical power and should be interpreted cautiously. In the EMAP subgroup, functional stability was observed without evidence of consistent improvement. Visual field mean deviation and OCT findings were consistent with absence of short-term deterioration across available paired data. Electrophysiological outcomes showed no consistent directional change. **Conclusions:** Repeated subtenon PRP administration appeared feasible and well tolerated in this small, uncontrolled pilot cohort. Any observed functional changes are preliminary and hypothesis-generating only and do not establish efficacy. Larger, adequately powered controlled studies with standardized endpoints are required to determine the potential role of PRP in degenerative retinal diseases.

## 1. Introduction

Retinal degenerative diseases represent a major cause of irreversible visual impairment and encompass a heterogeneous group of conditions characterized by progressive photoreceptor dysfunction and loss. Among these, retinitis pigmentosa (RP) represents the most common inherited retinal dystrophy, while extensive macular atrophy with pseudodrusen-like appearance (EMAP) has emerged as a distinct and particularly aggressive degenerative macular phenotype. Despite differences in etiology and clinical presentation, both conditions ultimately converge toward progressive retinal dysfunction, inflammation, and substantial visual disability, for which broadly applicable disease-modifying therapies remain limited [1,2].

Retinitis pigmentosa is a genetically and phenotypically heterogeneous group of inherited retinal dystrophies marked by progressive degeneration of photoreceptors, typically beginning with rod dysfunction and followed by secondary cone involvement [1]. Clinically, this process manifests as nyctalopia, progressive visual field constriction, and eventual impairment of central vision in advanced stages. Importantly, photoreceptor dysfunction in RP often precedes irreversible cell loss, creating a potential therapeutic window in which neuroprotective or modulatory interventions may enhance retinal function. Although recent advances in gene-specific therapies, optogenetics, and cell-based approaches have expanded treatment options for selected molecular subtypes, the majority of patients still lack therapeutic strategies capable of broadly modifying disease progression across diverse genotypes [2,3].

In contrast, EMAP is a distinct degenerative macular disorder characterized by early-onset bilateral macular atrophy, pseudodrusen-like deposits extending beyond the posterior pole, progressive loss of the outer retina and retinal pigment epithelium, and marked chorioretinal thinning [3,4]. Unlike classical inherited retinal dystrophies, EMAP primarily affects the macula and follows a rapid and highly predictable course toward severe central vision loss. Multimodal imaging studies have demonstrated extensive involvement of the photoreceptor–RPE–Bruch’s membrane complex, progressive enlargement of atrophic areas, and significant functional decline [4,5,6,7]. In this context, the early and extensive structural damage results in a limited residual functional substrate and reduced capacity for measurable functional recovery.

Increasing experimental and clinical evidence suggests that both RP and EMAP are driven not only by primary degenerative mechanisms but also by sustained para-inflammatory and immune-mediated processes at the level of the outer retina, retinal pigment epithelium, and choroid [8,9]. Dysregulation of microglial activity, oxidative stress, complement activation, and chronic release of inflammatory mediators contribute to secondary neuronal damage, accelerate photoreceptor loss, and amplify structural and functional decline in degenerative retinal diseases [8,9,10,11,12,13,14]. In this context, inflammatory cytokines, including tumor necrosis factor-alpha (TNF-α), have been implicated as key modulators of retinal neurodegeneration [10,11,12]. These insights support the rationale for biologic therapies capable of modulating the retinal microenvironment as a complementary approach to gene-specific or cell-replacement strategies.

Within this biological framework, autologous platelet-rich plasma (PRP) has emerged as a potential multimodal biologic therapy in regenerative medicine. PRP is a concentrated suspension of platelets in plasma, enriched with endogenous growth factors and cytokines, including platelet-derived growth factor, transforming growth factor-beta, insulin-like growth factor-1, and basic fibroblast growth factor [15,16,17,18]. Collectively, these factors may support retinal homeostasis by modulating inflammatory signaling, enhancing chorioretinal perfusion, stabilizing the extracellular matrix, and promoting cellular survival pathways within the neuroretina and retinal pigment epithelium.

In ophthalmology, PRP has been explored as a regenerative adjunct in several degenerative and inflammatory ocular conditions. Notably, studies investigating subtenon autologous PRP in retinitis pigmentosa have reported functional stabilization and, in selected cases, modest improvements in visual acuity, visual field sensitivity, and electrophysiological parameters [19,20,21,22]. These findings suggest that PRP may act primarily as a biologic modulator of the degenerative retinal microenvironment, rather than a curative intervention, particularly in diseases where residual viable retinal cells and functional plasticity persist.

The subtenon route of administration offers a minimally invasive approach that allows gradual and sustained diffusion of PRP-derived bioactive factors toward the posterior segment, including the choroid, retinal pigment epithelium, and outer retina, while minimizing the procedural risks associated with intravitreal delivery [17,18]. This approach is particularly suitable for chronic degenerative retinal diseases, in which safety, repeatability, and long-term tolerability are critical considerations. However, current clinical evidence remains limited and primarily exploratory, with a lack of adequately powered controlled studies.

Accordingly, this study was designed as a prospective, open-label pilot investigation primarily focused on safety and feasibility, with exploratory assessment of functional and structural outcomes in patients with retinitis pigmentosa and EMAP. By integrating multimodal functional assessments and structural imaging biomarkers, this study aims to generate preliminary, hypothesis-generating data to inform the design of future controlled trials in degenerative retinal diseases characterized by progressive photoreceptor loss and macular atrophy. Importantly, this study was designed exclusively to evaluate safety and feasibility, and not to test efficacy. Any functional or structural observations should therefore be interpreted as exploratory and hypothesis-generating [23].

## 2. Materials and Methods

### 2.1. Study Design and Ethics

This study was designed as a prospective, open-label, exploratory pilot clinical study primarily aimed at evaluating the safety and feasibility of repeated subtenon administration of autologous platelet-rich plasma (PRP) in patients with degenerative retinal diseases, specifically retinitis pigmentosa (RP) and extensive macular atrophy with pseudodrusen-like appearance (EMAP).

Exploratory analyses of functional and structural outcomes were performed to generate preliminary signals to inform future controlled studies. The study was not designed or powered to evaluate efficacy.

All analyses were conducted per patient, using a single study eye per participant. The study eye was predefined as the eye with better baseline best-corrected visual acuity (BCVA) when both eyes were eligible. In cases of similar BCVA between eyes, the eye with more reliable and complete baseline functional assessments, including visual field and OCT data, was selected. This predefined selection rule was applied consistently across all participants to minimize potential selection bias.

The study was conducted in accordance with the Declaration of Helsinki and national regulations for research involving human subjects. Ethical approval was obtained from the Brazilian National Research Ethics Committee (CONEP) (CAAE: 77066424.0.0000.0317). The clinical protocol was registered at ClinicalTrials.gov (Identifier: NCT07341919; RETINA-PRP Study; Registration Date: 1 February 2024). Written informed consent was obtained from all participants prior to enrollment.

### 2.2. Study Population and Eligibility Criteria

#### 2.2.1. Inclusion Criteria

Participants were eligible if they met all of the following criteria:

- Age ≥ 18 years
- Diagnosis of RP and EMAP was established based on multimodal retinal imaging, including spectral-domain OCT, fundus autofluorescence (FAF), and color fundus photography. RP diagnosis relied on characteristic clinical features such as bone-spicule pigmentation, retinal vascular attenuation, and optic disc pallor, supported by electrophysiological findings when available. EMAP diagnosis required the presence of bilateral macular atrophy associated with pseudodrusen-like deposits and chorioretinal thinning on multimodal imaging. No predefined staging or severity thresholds were applied, given the exploratory nature of this pilot study.
- Best-corrected visual acuity (BCVA) of counting fingers at 1 m or better (≤1.9 logMAR) in the study eye
- Measurable visual field using iCare COMPASS automated perimetry with acceptable reliability indices
- Clear ocular media allowing safe periocular injection and adequate OCT imaging
- Ability and willingness to comply with study visits

For the electrophysiology subgroup, a recordable baseline 30-Hz flicker ERG response (signal-to-noise ratio ≥ 3:1 and amplitude ≥ 3.0 µV) was required; absence of a measurable response did not preclude inclusion in the main analysis.

#### 2.2.2. Exclusion Criteria

Exclusion criteria included:

- Active ocular inflammation or infection
- Active choroidal neovascularization or other unrelated macular diseases
- Uncontrolled glaucoma (IOP > 21 mmHg despite treatment)
- Significant media opacity affecting imaging or injection safety
- Recent ocular interventions (intravitreal therapy, periocular steroid injection, or intraocular surgery within 3 months)
- Coagulopathy or contraindication to periocular injection (platelets < 100,000/µL or INR > 1.5)
- Pregnancy or breastfeeding
- Participation in another interventional clinical trial within 3 months

### 2.3. PRP Preparation

Autologous PRP was prepared under standardized sterile conditions using a closed double-spin centrifugation system, ensuring reproducibility and minimizing contamination risk. Peripheral venous blood (30–60 mL) was collected in sodium citrate tubes, with an additional EDTA tube for baseline platelet count. The preparation involved a first centrifugation at 300× *g* for 10 min, followed by a second centrifugation at 640× *g* for 10 min. The final product consisted of leukocyte-poor PRP, with a mean platelet concentration of 735 ± 265 × 10^3^/µL, corresponding to approximately 2.9-fold enrichment relative to baseline. No exogenous activation agents were used, and the mean time between blood collection and injection was approximately 2 h. Microbiological sterility testing was performed prior to administration to confirm absence of contamination. All PRP preparations met predefined release criteria, including sterility and target platelet enrichment. Leukocyte depletion was not quantitatively measured but was controlled by the standardized preparation protocol. The same processing protocol was used for all patients to ensure consistency.

### 2.4. Intervention Protocol

Participants received subtenon injections of autologous PRP (1.5 mL per injection) administered in the inferotemporal quadrant under topical anesthesia. The treatment protocol consisted of three injections at baseline (Month 0), Month 2 (M2), and Month 4 (M4). As a precaution to reduce the risk of transient intraocular pressure elevation, oral acetazolamide (500 mg) was administered 1 h prior to injection. All injections were performed by the same experienced retinal specialist to ensure procedural consistency. Laterality was determined based on predefined study-eye selection criteria. No topical or systemic post-injection medications were routinely prescribed, except for prophylactic acetazolamide. The injection volume (1.5 mL) and the 2-month interval were selected based on prior exploratory clinical experience and safety considerations to balance biological exposure and tolerability. The subtenon route was chosen to allow gradual diffusion of PRP-derived bioactive factors toward the choroid, retinal pigment epithelium, and outer retina, while minimizing risks associated with intravitreal administration. No additional regenerative or biologic ocular therapies were permitted during the study period.

### 2.5. Clinical Assessments and Follow-Up

Participants underwent standardized evaluations at baseline and Month 6.

Safety visits were performed 7–14 days after each injection, including slit-lamp examination, fundus evaluation, and intraocular pressure (IOP) measurement.

Assessments included:

- Best-corrected visual acuity (BCVA, logMAR)
- Automated visual field testing (Mean Deviation, MD)
- Spectral-domain OCT (central macular thickness and qualitative ellipsoid zone assessment)
- Intraocular pressure (IOP)
- Optional 30-Hz flicker ERG

### 2.6. Outcome Measures

The primary outcome of this study was safety and tolerability, including the assessment of ocular adverse events, intraocular pressure changes, and inflammatory responses associated with repeated subtenon PRP administration.

Secondary outcomes were defined as exploratory and included functional and structural parameters. These comprised best-corrected visual acuity (BCVA), expressed in logMAR, visual field mean deviation (MD, measured in decibels), and structural optical coherence tomography (OCT) parameters, including central subfield thickness (CST) and qualitative assessment of ellipsoid zone (EZ) integrity.

Electrophysiological outcomes, specifically 30-Hz flicker electroretinography (ERG), were considered ancillary descriptive measures and were analyzed descriptively due to incomplete paired data and variability in signal detectability across participants (Table 1).

### 2.7. Missing Data and Data Availability

Due to the exploratory nature of the study and real-world clinical variability, not all endpoints were available for all participants. Missing data were primarily related to advanced disease stage, poor fixation, or unreliable test performance. Missingness was not systematically different between RP and EMAP but was more frequent in patients with advanced functional impairment. Patients with complete paired data did not differ significantly in baseline BCVA from those with incomplete data. Analyses were therefore performed using available paired data for each endpoint.

### 2.8. Statistical Analysis

Continuous variables are presented as mean ± standard deviation. Normality of paired differences was assessed using the Shapiro–Wilk test. Depending on data distribution, paired comparisons were performed using either the paired *t*-test or the Wilcoxon signed-rank test.

Given the small sample size, all statistical analyses should be interpreted as exploratory. Effect sizes (mean differences) and corresponding 95% confidence intervals are reported when applicable. Subgroup analyses (RP and EMAP) were conducted descriptively and are intended for hypothesis generation only. *p*-values are presented as nominal and were not adjusted for multiple comparisons. A two-sided *p*-value < 0.05 was considered nominally significant.

## 3. Results

### 3.1. Study Population

A total of 13 patients with degenerative retinal diseases were included, comprising 6 patients with retinitis pigmentosa (RP) and 7 patients with extensive macular atrophy with pseudodrusen-like appearance (EMAP). All participants completed the planned treatment protocol of three subtenon injections of autologous platelet-rich plasma (PRP) and had at least one post-treatment follow-up visit.

Paired best-corrected visual acuity (BCVA) data were available for all 13 patients, paired visual field data for 9 patients, and structural OCT data for 8 patients. Electrophysiological data were available only for a subset of patients.

**Figure 1:**
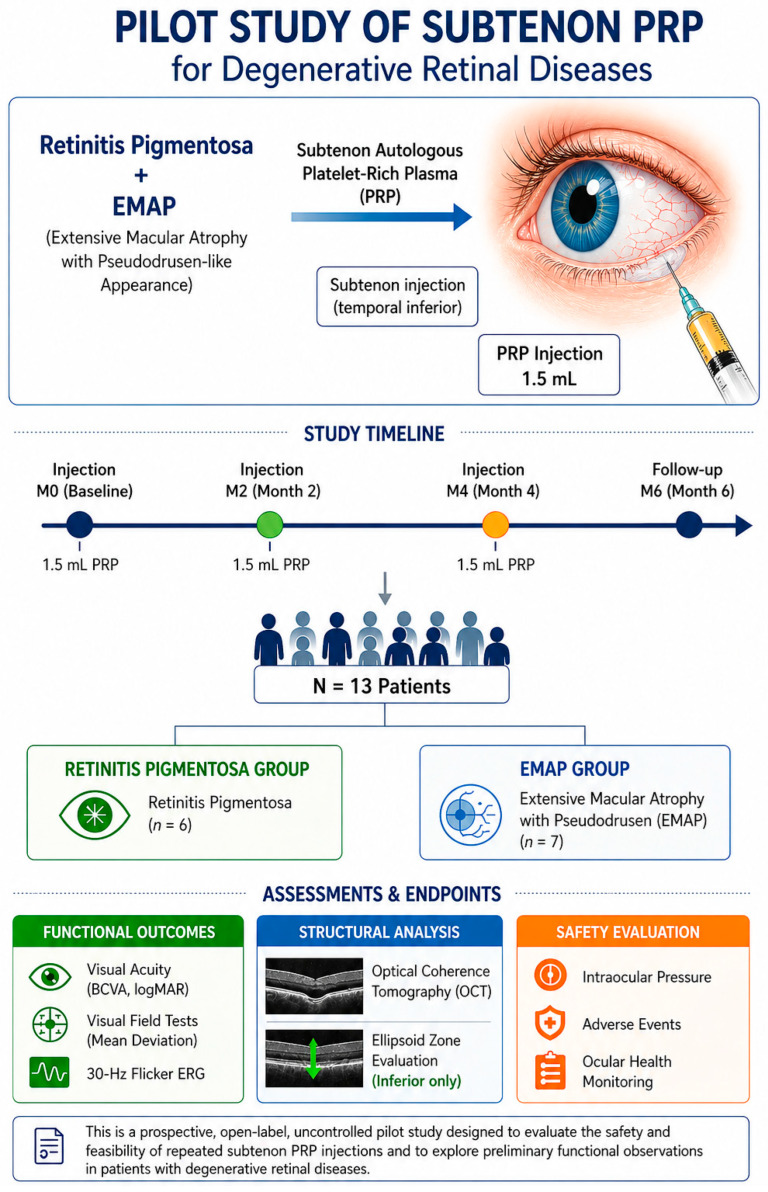
Study design of a prospective, open-label, uncontrolled pilot study evaluating the safety and feasibility of subtenon autologous platelet-rich plasma (PRP) in degenerative retinal diseases, including retinitis pigmentosa (RP) and extensive macular atrophy with pseudodrusen-like appearance (EMAP). All participants received three subtenon PRP injections (1.5 mL each) at baseline (Month 0), Month 2 (M2), and Month 4 (M4), with functional and structural assessments performed through Month 6 (M6).

The study design and follow-up schedule are summarized in Figure 1.

### 3.2. Best-Corrected Visual Acuity

**Figure 2:**
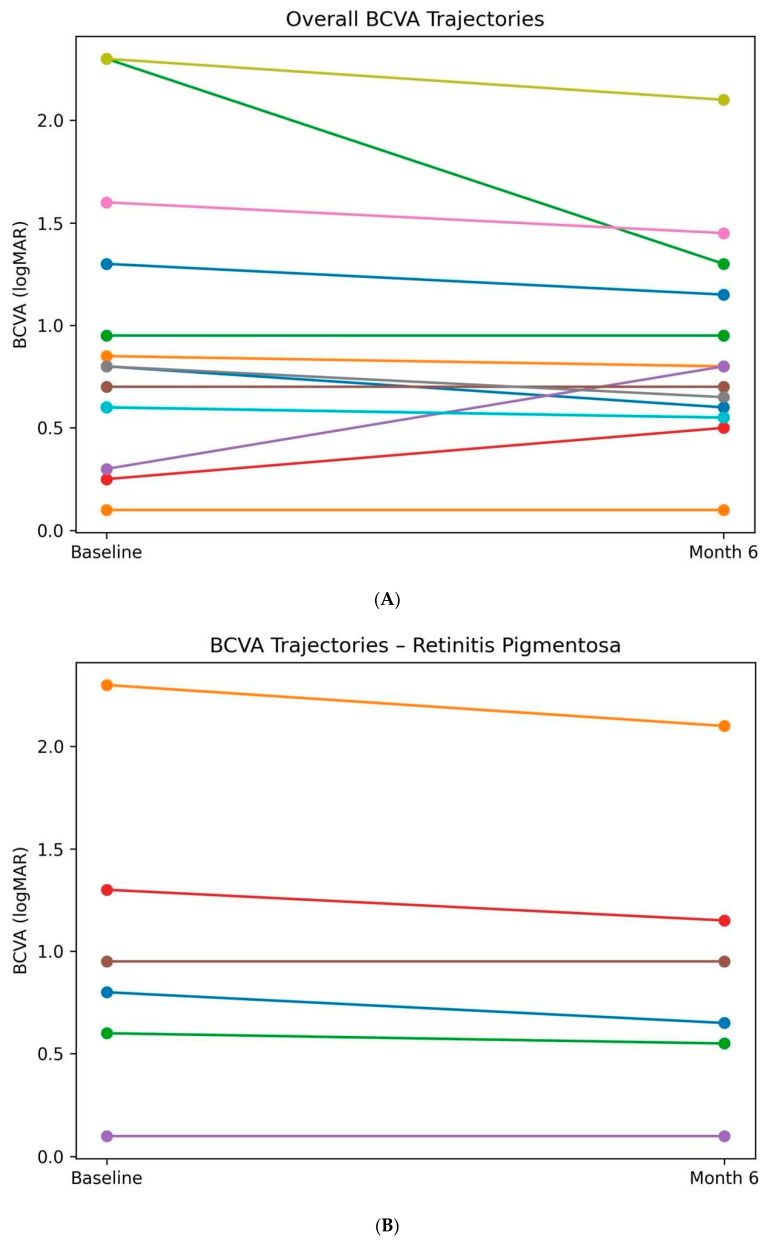

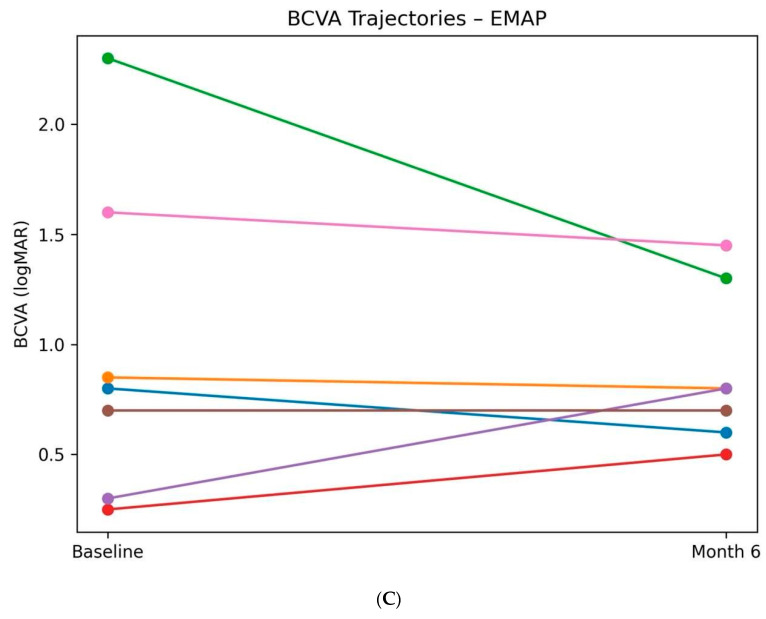
(**A**) Individual pre–post trajectories (spaghetti plot) of best-corrected visual acuity (BCVA), expressed in logMAR, in the overall cohort following subtenon autologous platelet-rich plasma (PRP) injections. Each line represents one patient, illustrating individual variability and overall functional trends from baseline to Month 6. Individual values and subgroup sample sizes are displayed to illustrate variability and uncertainty. (**B**) Individual pre–post trajectories of best-corrected visual acuity (BCVA, logMAR) in patients with retinitis pigmentosa (RP) treated with subtenon autologous platelet-rich plasma. Most patients demonstrated functional stability or mild improvement over the 6-month follow-up period. Individual values and subgroup sample sizes are displayed to illustrate variability and uncertainty. (**C**) Individual pre–post trajectories of best-corrected visual acuity (BCVA, logMAR) in patients with extensive macular atrophy with pseudodrusen-like appearance (EMAP) treated with subtenon autologous platelet-rich plasma. Visual acuity remained predominantly stable, consistent with the aggressive atrophic nature of the disease. Individual values and subgroup sample sizes are displayed to illustrate variability and uncertainty.

In the overall cohort, mean BCVA showed a small, non-significant change from 0.99 ± 0.71 logMAR at baseline to 0.90 ± 0.51 logMAR at Month 6 (*p* = 0.283) (Figure 2A–C).

When analyzed within diagnostic subgroups, patients with retinitis pigmentosa (RP) demonstrated a modest improvement in BCVA (mean ΔlogMAR − 0.09), corresponding to approximately 4–5 ETDRS letters. When analyzed within diagnostic subgroups, patients with retinitis pigmentosa (RP) demonstrated a modest change in BCVA (mean ΔlogMAR − 0.09), corresponding to approximately 4–5 ETDRS letters. This change reached nominal statistical significance (*p* = 0.048) but was associated with a small effect size and wide confidence intervals. This nominal finding should be interpreted cautiously given the small sample size, wide confidence intervals, and absence of multiplicity correction. Accordingly, this observation should be considered exploratory and hypothesis-generating only.

In contrast, patients with EMAP demonstrated functional stability, with mean BCVA changing from 0.97 ± 0.74 logMAR at baseline to 0.88 ± 0.36 logMAR at Month 6 (*p* = 0.619), without evidence of consistent functional improvement.

Between-group comparisons of BCVA change were not performed as efficacy comparisons were not a predefined objective of this pilot study.

**Figure 3:**
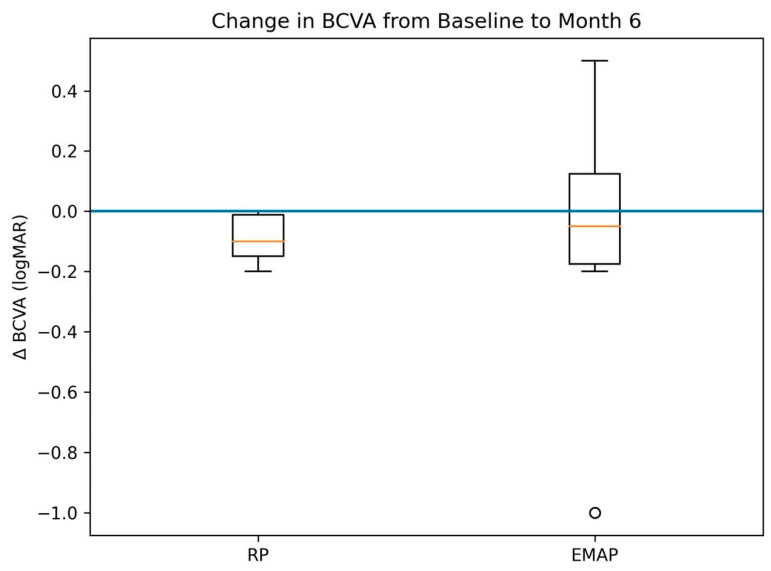
Boxplot showing the distribution of changes in best-corrected visual acuity (ΔlogMAR) from baseline to Month 6 in patients with retinitis pigmentosa and EMAP. Negative values indicate improvement in visual acuity. The horizontal dashed line represents no change from baseline. Individual values and subgroup sample sizes are displayed to illustrate variability and uncertainty.

Individual BCVA trajectories (Figure 2A–C and Figure 3) demonstrated substantial inter-individual variability, with a predominance of functional stability and mild improvement, particularly in the RP subgroup.

### 3.3. Visual Field Outcomes

Paired automated visual field data were available for 9 of the 13 patients. Due to variability in testing strategies (10-2 and 24-2) and incomplete paired data, visual field analysis was standardized using mean deviation (MD, dB) as the primary metric.

In the overall cohort, MD showed a small, non-significant change from −10.83 ± 8.78 dB at baseline to −10.23 ± 8.69 dB at Month 6 (*p* = 0.352), consistent with functional stability over the follow-up period.

Given the limited sample size, heterogeneity of perimetric strategies, and missing data, subgroup analyses were considered exploratory and are presented descriptively. No consistent pattern of visual field improvement was observed.

**Figure 4:**
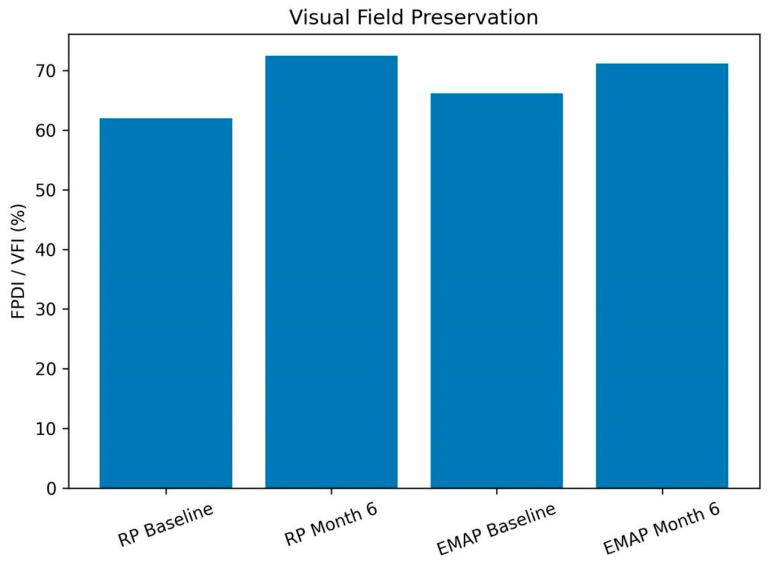
Visual field outcomes before and after subtenon autologous platelet-rich plasma injections, stratified by diagnosis. Bars represent mean values of Mean Deviation (MD, dB) at baseline and Month 6, derived from automated perimetry (iCare COMPASS). Individual values and subgroup sample sizes are displayed to illustrate variability and uncertainty. No consistent pattern of functional improvement was observed, and findings are presented as exploratory.

Visual field trajectories are summarized in Figure 4, demonstrating relative stability across the cohort without evidence of progressive deterioration during the study period.

These findings should be interpreted cautiously within the exploratory framework of this pilot study and do not support conclusions regarding treatment efficacy.

### 3.4. Structural Outcomes (OCT Analysis)

Paired OCT data were available for 8 of the 13 patients and were analyzed as a predefined exploratory subset. All OCT scans were acquired using the same spectral-domain OCT device and a standardized acquisition protocol across all visits, ensuring consistency in structural assessment.

#### 3.4.1. Central Subfield Thickness (CST)

In the EMAP subgroup (n = 4), mean CST decreased from 231.8 ± 16.5 µm to 218.5 ± 13.0 µm (mean change −13.3 µm), without evidence of intraretinal fluid or reactive thickening, consistent with structural stability.

In the RP subgroup (n = 4), CST showed greater inter-individual variability, with a mean increase from 214.3 ± 58.3 µm to 258.5 ± 68.4 µm. Given the high variability and small sample size, these findings should be interpreted as exploratory.

#### 3.4.2. Ellipsoid Zone (EZ) Integrity

Ellipsoid zone integrity was assessed qualitatively and graded by a single experienced evaluator, not masked to timepoint, and categorized as preserved, disrupted, or absent.

In the EMAP subgroup, all eyes maintained their baseline classification (absent or fragmented EZ), indicating no structural worsening and no evidence of regeneration.

In the RP subgroup, 2 of 4 eyes demonstrated qualitative improvement in EZ reflectivity, although without full anatomical restoration. No eye showed progression to a worse category.

Overall, PRP was not associated with overt structural regeneration; however, structural stability was observed in EMAP, and a subtle qualitative signal was noted in selected RP cases.

### 3.5. Electrophysiological Outcomes

Electrophysiological assessment using 30-Hz flicker ERG was limited by incomplete paired data and low baseline amplitudes in advanced disease stages. Paired ERG data were available in a limited subset of patients. Descriptive analysis showed no evidence of consistent deterioration or loss of measurable responses over the follow-up period. Given these limitations, ERG findings are reported as exploratory and should not be used to infer treatment efficacy.

### 3.6. Safety and Adverse Events

No serious adverse events or permanent ocular morbidity were observed during the study period. Adverse events were recorded prospectively at each visit and classified by severity and relationship to treatment. Two transient and manageable ocular adverse events were recorded:

- One case of mild anterior uveitis in a patient with prior HLA-B27–associated uveitis, which resolved with topical corticosteroid therapy
- One case of acute intraocular pressure elevation (40 mmHg) in an EMAP patient, attributed to an angle-closure mechanism and successfully managed without long-term sequelae

No cases of infectious complications, sustained inflammation, cystoid macular edema (CME), or choroidal neovascularization (CNV) were observed. Overall, subtenon PRP demonstrated a favorable short-term safety profile, with adverse events being infrequent, transient, and manageable.

## 4. Discussion

In this prospective pilot study, subtenon autologous platelet-rich plasma (PRP) was primarily evaluated in terms of safety and feasibility rather than efficacy. The findings indicate that repeated subtenon PRP administration is well tolerated in patients with degenerative retinal diseases, with no evidence of treatment-related structural deterioration over short-term follow-up.

An exploratory functional observation was noted in the retinitis pigmentosa (RP) subgroup, particularly in best-corrected visual acuity, although the magnitude of change was small and associated with limited statistical power. This finding may suggest a potential exploratory observation in conditions with residual retinal viability, rather than evidence of a treatment effect. In contrast, patients with extensive macular atrophy with pseudodrusen-like appearance (EMAP) demonstrated functional stability without measurable improvement, which may reflect the advanced structural damage characteristic of this condition. These findings should be interpreted as hypothesis-generating and do not establish treatment efficacy.

### 4.1. Differential Biological Substrate in RP and EMAP

Retinitis pigmentosa is characterized by progressive photoreceptor degeneration, initially affecting rods and subsequently cones, while portions of the inner retinal architecture and retinal pigment epithelium (RPE) may remain structurally preserved for extended periods, particularly in earlier disease stages [1,2]. Importantly, photoreceptor dysfunction may precede irreversible cell loss, creating a potential therapeutic window in which neuroprotective or immunomodulatory interventions may influence functional performance.

In contrast, EMAP is defined by early-onset bilateral macular atrophy, extensive involvement of the outer retina, RPE, Bruch’s membrane, and choriocapillaris, and a rapid course toward severe central vision loss [3,4,5,6]. In this context, the limited residual functional substrate constrains the potential for measurable short-term functional improvement. Consequently, biologic modulation strategies are more likely to result in stabilization rather than recovery in EMAP.

### 4.2. Mechanistic Rationale for PRP Effects

PRP contains a mixture of growth factors and cytokines, including platelet-derived growth factor (PDGF), transforming growth factor-beta (TGF-β), insulin-like growth factor-1 (IGF-1), and vascular endothelial growth factor (VEGF), which may exert neurotrophic, anti-inflammatory, and immunomodulatory effects [15,16,17,18]. These factors may influence retinal homeostasis by modulating inflammatory signaling, enhancing cellular metabolism, and supporting photoreceptor survival within the degenerative retinal microenvironment.

In RP, chronic para-inflammatory processes, oxidative stress, mitochondrial dysfunction, and microglial activation are recognized contributors to disease progression [8,9,10,11]. Modulation of these pathways may influence the functional performance of surviving photoreceptors without necessarily inducing structural regeneration. The modest change observed in BCVA in the RP subgroup, despite limited structural variation, is consistent with this hypothesis, although it remains exploratory.

### 4.3. Functional Stability in EMAP

The absence of measurable functional improvement in EMAP likely reflects advanced degeneration of the outer retina and RPE, where the primary cellular targets of PRP-mediated modulation are substantially reduced or absent [3,4,5,6]. In this setting, biologic modulation may not translate into short-term improvements detectable by conventional functional endpoints.

These findings may reflect differences in underlying disease biology and residual retinal viability. The absence of deterioration observed in this cohort may represent a possible stabilization, requiring confirmation in controlled studies with longer follow-up and standardized endpoints.

### 4.4. Functional–Structural Dissociation

The dissociation observed between functional outcomes (BCVA) and structural or electrophysiological measures is consistent with early-phase biologic investigations. Functional changes may reflect variations in retinal efficiency, synaptic activity, or metabolic support rather than increases in photoreceptor number or structural regeneration. Electrophysiological measures such as 30-Hz flicker ERG may have limited sensitivity in advanced disease stages, particularly in small cohorts with low baseline amplitudes. Therefore, the absence of measurable ERG changes should not be interpreted as evidence of lack of biological activity.

**Figure 5:**
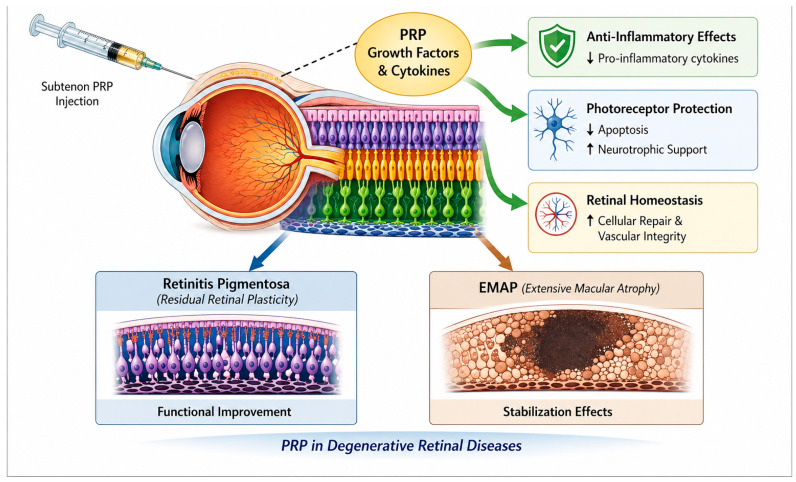
Proposed mechanistic model of subtenon autologous platelet-rich plasma (PRP) in degenerative retinal diseases. PRP-derived growth factors and cytokines may modulate inflammatory signaling, support photoreceptor survival, and enhance retinal homeostasis. Functional benefits are more evident in conditions with residual retinal plasticity, such as retinitis pigmentosa, whereas stabilizing effects predominate in aggressive macular atrophic phenotypes such as EMAP.

The proposed biological rationale underlying these observations is summarized schematically in Figure 5, which illustrates a conceptual model in which subtenon PRP delivers growth factors and cytokines that may modulate inflammatory signaling, support photoreceptor survival pathways, and contribute to retinal homeostasis. This model is derived from prior literature and is presented as a hypothesis-generating framework rather than direct mechanistic evidence from the present dataset.

### 4.5. Safety Considerations

Subtenon PRP demonstrated a favorable short-term safety profile, with no serious adverse events or permanent ocular morbidity observed. The two adverse events reported were transient and manageable: one episode of mild anterior uveitis in a predisposed patient and one episode of acute intraocular pressure elevation associated with an angle-closure mechanism.

The relatively large subtenon injection volume (1.5 mL) may transiently increase orbital pressure, which should be considered in patients with predisposing anatomical factors. Careful patient selection and monitoring remain essential. The absence of treatment-emergent cystoid macular edema, choroidal neovascularization, or structural worsening on OCT further supports the short-term tolerability of repeated subtenon PRP administration.

### 4.6. Limitations and Future Directions

This study has several important limitations, including the small sample size, absence of a control group, heterogeneity of disease stage, incomplete data across endpoints, and short follow-up duration, all of which limit the interpretation of outcomes. Additional limitations include potential regression to the mean, learning effects in repeated functional testing, and the risk of selective emphasis on nominally significant findings. The exploratory nature of the analyses and the absence of adjustment for multiple comparisons further require cautious interpretation.

This pilot study was designed to generate preliminary data to inform future investigations. The findings support feasibility and short-term safety, while any functional observations remain exploratory. Future studies should prioritize adequately powered randomized controlled designs, standardized functional endpoints, quantitative structural biomarkers, longer follow-up durations, and stratification by disease phenotype and stage.

## 5. Conclusions

Subtenon autologous platelet-rich plasma (PRP) demonstrated a favorable short-term safety and tolerability profile in this prospective pilot study of degenerative retinal diseases. An exploratory observation was noted in patients with retinitis pigmentosa, whereas patients with extensive macular atrophy with pseudodrusen-like appearance (EMAP) showed maintenance of function without evidence of accelerated deterioration over the follow-up period. These findings may reflect differences in underlying disease biology and residual retinal viability.

Importantly, this study was not designed to evaluate efficacy, and the results should be interpreted as hypothesis-generating. The observed patterns may represent possible stabilization, requiring confirmation in adequately powered, controlled clinical trials with standardized functional and structural endpoints.

## Figures and Tables

**Table 1:** Availability of paired data across functional and structural endpoints at baseline and Month 6. This table summarizes the number of patients with available paired data for each study endpoint, including best-corrected visual acuity (BCVA), visual field mean deviation (MD), optical coherence tomography (OCT), and electrophysiological assessment (ERG). Due to the exploratory design and advanced disease stage, data availability varied across endpoints, particularly for visual field and OCT analyses. ERG measurements were available only in a limited subset of patients due to reduced signal detectability in advanced retinal degeneration.

Endpoint	Baseline (*n*)	Follow-Up (*n*)
BCVA	13	13
Visual field	13	9
OCT	13	8
ERG	subset	subset

## Data Availability

The data presented in this study are available from the corresponding author upon reasonable request. The data are not publicly available due to ethical and privacy restrictions involving human participants.
